# Supplementary material for: Using Microcystin Gene Copies to Determine Potentially-Toxic Blooms, Example from a Shallow Eutrophic Lake Peipsi
Source: Toxins (Basel). 2020 Mar 26;12(4):211. doi: 10.3390/toxins12040211 (PMC7232469; doi:10.3390/toxins12040211)
Supplement: Supplementary file 1 [file toxins-12-00211-s001.zip › supp/toxins-721320 supp. for proofreading_KP.docx]

Supplementary Materials: Using Microcystin Gene Copies to Determine Potentially-Toxic Blooms, Example from a Shallow Eutrophic Lake Peipsi

Kristel Panksep, Marju Tamm, Evanthia Mantzouki, Anne Rantala-Ylinen, Reet Laugaste,
Kaarina Sivonen, Olga Tammeorg and Veljo Kisand

**Table S2.** Coordinates of the sampling points.

| **Sampling Point** | **Basin** | **N** | **E** |
| --- | --- | --- | --- |
| 2 | Peipsi *s.s.* | 58˚50'04'' | 27˚06'25'' |
| 4 | Peipsi *s.s.* | 58˚48'34'' | 27˚22'18'' |
| 5 | Peipsi *s.s.* | 58˚47'17'' | 27˚31'50'' |
| 7 | Peipsi *s.s.* | 58˚46'20'' | 27˚43'47'' |
| 10 | Peipsi *s.s.* | 58˚43'57'' | 27˚26'08'' |
| 91 | Peipsi *s.s.* | 58˚35'02'' | 27˚38'01'' |
| 11 | Peipsi *s.s.* | 58˚35'12'' | 27˚26'12'' |
| 38 | Peipsi *s.s.* | 58˚26'36'' | 27˚16'36'' |
| 43 | Peipsi *s.s.* | 58˚25'33'' | 27˚36'55'' |
| 56 | Peipsi *s.s.* | 58˚19'24'' | 27˚36'11'' |
| 16 | Lämmijärv | 58˚14'02'' | 27˚29'12'' |
| 17 | Lämmijärv | 58˚07'12'' | 27˚34'30'' |
| 51 | Pihkva | 58˚05'21'' | 27˚53'39'' |
| 27 | Pihkva | 58˚05'39'' | 27˚41'19'' |
| 52 | Pihkva | 57˚59'21'' | 27˚59'38'' |
| 22 | Pihkva | 57˚54'39'' | 28˚06'01'' |

**Table S3.** Sequences of the primers and hydrolysis probe designed to detect and quantify *Planktothrix mcyE* genes in environmental samles by quantitative real-time PCR assay .

| Primer, Probe | Sequence |
| --- | --- |
| Forward 664F | 5’- CATTGCCGGATTAGGCGT-3’ |
| Reverse 744R | 5’- AGGATTTTTGCAGAGGAATTGTG-3’ |
| Probe 670P | 5’-TAAAAGTGAATATGATTATCAAGAACCCATCCCC-3’ |

**Table S4.** Average threshold-cycle values (SD) of three replicate reactions, obtained with cyanobacterial strains used to test the specificity of the genus-specific *Planktothrix-mcyE* gene quantitative real-time PCR assay.

| **Strain** | ***Planktothrix mcyE*** | **Hepatotoxin Production^c^** |
| --- | --- | --- |
| *Planktothrix* spp. |  |  |
| 49 | 21.6 (0.431) | mc |
| 97 | 23.0 (0.287) | mc |
| NIVA-CYA126/8 | 20.0 (0.222) | mc |
| NIVA-CYA127 | 20.28 (0.525) | mc |
| NIVA-CYA128/R | 20.38 (0.081) | mc |
| P213 | 24.2 (0.611) | mc |
| P226 | 23.9 (0.711) | mc |
| P214 | ND^*^ | - |
| P45 | ND | - |
| P6304 | 34.9 (0.711) | - |
| P18 | ND | - |
| P7515 | ND | - |
| P2 | ND | - |
| *Microcystis* spp. |  | - |
| PCC 7806 | ND | - |
| PCC 7941 |  |  |
| *Dolichospermum* spp. |  |  |
| 90 | ND | mc |
| 202A1 | ND | mc |
| *Nodularia* spp. |  |  |
| HEM | ND | nod |
| BY1 | ND | nod |
| *Nostoc* spp. |  |  |
| 152 | ND | mc |
| IO-102.1 | ND | mc |

* ND, not detected or threshold-cycle value under detection limit (10 copies of *mcyE* gene in a reaction).


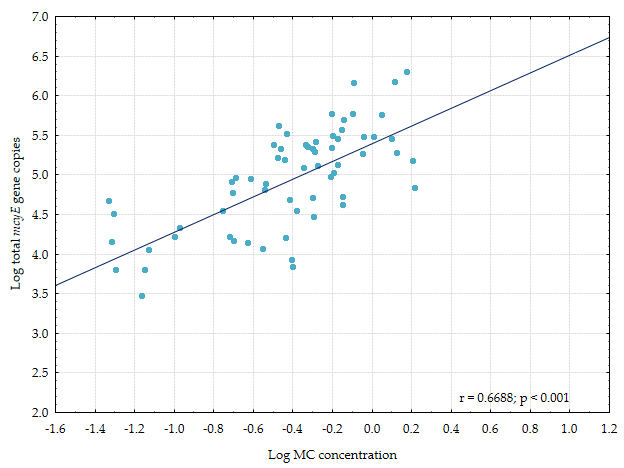


**Figure S1.** Relationship between MC concentration and the sum of *Microcystis, Dolichospermum* and *Planktothrix mcyE* gene copy numbers.


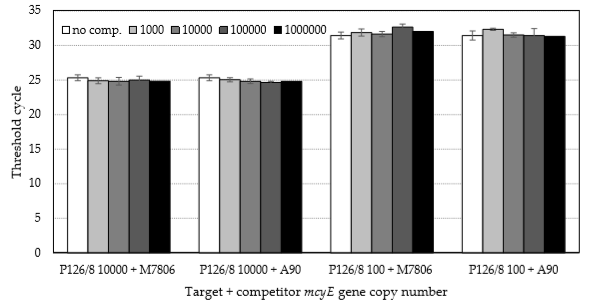


**Figure S2.** Average threshold cycles and standard deviations of triplicate *Planktothrix mcyE* targeted by qPCR assays. Assays were performed either with 10^4^ or 10^2^ copies of target mcyE gene and 0, 10^3^, 10^4^, 10^5^, or 10^6^ copies of competing *mcyE* genes of two other microcystin-producing strains. Strain abbreviations: P126/8—*Planktothrix agardhii* 126/8; A90—*Dolichospermum* sp. 90 and M7806—*Microcystis aeruginosa*. PCC 7806.
